# ReactomeFIViz: a Cytoscape app for pathway and network-based data analysis

**DOI:** 10.12688/f1000research.4431.2

**Published:** 2014-09-12

**Authors:** Guanming Wu, Eric Dawson, Adrian Duong, Robin Haw, Lincoln Stein

**Affiliations:** 1 Ontario Institute for Cancer Research, Toronto, Ontario M5G 0A3, Canada; 2 DMICE, Oregon Health & Science University, Portland, Oregon 97239, USA; 3 Section of Integrative Biology, Institute for Cellular and Molecular Biology, and Center for Computational Biology and Bioinformatics, The University of Texas at Austin, Austin, TX 78712, USA; 4 Department of Molecular Genetics, University of Toronto, Toronto, Ontario M5S 1A8, Canada

## Abstract

High-throughput experiments are routinely performed in modern biological studies. However, extracting meaningful results from massive experimental data sets is a challenging task for biologists. Projecting data onto pathway and network contexts is a powerful way to unravel patterns embedded in seemingly scattered large data sets and assist knowledge discovery related to cancer and other complex diseases. We have developed a Cytoscape app called “ReactomeFIViz”, which utilizes a highly reliable gene functional interaction network combined with human curated pathways derived from Reactome and other pathway databases. This app provides a suite of features to assist biologists in performing pathway- and network-based data analysis in a biologically intuitive and user-friendly way. Biologists can use this app to uncover network and pathway patterns related to their studies, search for gene signatures from gene expression data sets, reveal pathways significantly enriched by genes in a list, and integrate multiple genomic data types into a pathway context using probabilistic graphical models. We believe our app will give researchers substantial power to analyze intrinsically noisy high-throughput experimental data to find biologically relevant information.

## Introduction

High-throughput experiments, which generate large and complex data sets, are routinely performed in modern biological and clinical studies to unravel mechanisms underlying complex diseases, such as cancer. However, extracting reliable and meaningful results from these experiments is usually difficult and requires sophisticated computational tools and algorithms, which are challenging for experimental biologists to comprehend. A user-friendly software tool is extremely important for both bench and computational biologists to perform high-throughput data analysis related to cancer and other complex diseases.

Many studies have shown that alterations in pathways or networks are better correlated with complex disease phenotypes than any particular gene or gene product ^1, 2^. Pathway- and network-based data analysis approaches project information about seemingly unrelated genes and proteins onto pathway and network contexts, and create an integrated view for researchers to understand mechanisms related to phenotypes of interest.

In this paper, we describe a software tool called ReactomeFIViz (also called the Reactome FI Cytoscape app or ReactomeFIPlugIn), which can be used to perform pathway- and network-based data analysis for data generated from high-throughput experiments. This tool uses the highly reliable Reactome functional interaction (FI) network ^3^ for doing network-based data analysis. The FI network was constructed by merging interactions extracted from human curated pathways with interactions predicted using a machine learning approach. This tool can also be used to perform pathway-based data analysis by using high quality human-curated pathways in the Reactome database ^4^, the most comprehensive open source pathway database.

## Implementation

### Software architecture

**Figure 1.**
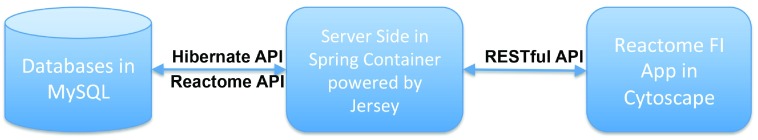
The three-tier software architecture used to implement ReactomeFIViz.

We used conventional three-tier software architecture to implement ReactomeFIViz ( Figure 1). The back-end contains several databases hosted in the open-source MySQL database engine ( http://www.mysql.com). The middle server-side application uses hibernate ( http://hibernate.org) to access the databases storing FIs and cancer gene index data (see below). The server-side application also uses the in-house developed Reactome API for Object/Relational mapping to access pathway-related contents stored in a database using the Reactome database schema. On the server-side, a lightweight servlet container, Spring Framework ( http://projects.spring.io/spring-framework/), and a Java RESTful framework, Jersey ( https://jersey.java.net), are used to power a RESTful API for the Cytoscape front-end. The front-end Cytoscape app uses this RESTful API to communicate with the server-side application. Almost all analysis features in the app are provided by this RESTful API, which should also facilitate their use by other front-end applications, such as a web browser or tablet app.

For cancer data analysis, we imported the cancer gene index (CGI, https://wiki.nci.nih.gov/display/cageneindex) data into a MySQL database and then developed a hibernate API for the server-side application. The CGI data contains annotations for cancer-related genes. These annotations were extracted by using text-mining technologies and then validated by human curators ( https://wiki.nci.nih.gov/display/cageneindex/Creation+of+the+Cancer+Gene+Index).

The Reactome FI network is updated annually. We recommend using the latest version of the FI network. Different versions of the FI network may yield different results due to updates to gene interactions, so we have also deployed two older versions of the FI network to use for comparison of legacy data sets and to reproduce published results.

R ( http://www.r-project.org) is used in the server-side for executing network module-based survival analysis and other statistical computations. ReactomeFIViz uses Java based methods in the server-side to call functions in R. Users of our app don’t need to install R in their machines in order to perform the statistical analyses implemented in the app.

ReactomeFIViz is designed and implemented for Cytoscape 3, and includes all features in Reactome FI Cytoscape plug-in for Cytoscape 2. Users are recommended to use the latest version of our app for Cytoscape 3.

### Network analysis features

ReactomeFIViz implements multiple features for users to perform network-based data analysis, including FI sub-network construction ^3^, network module discovery ^3^, functional annotation ^3^, HotNet mutation analysis ^5, 6^, and network module-based gene signature discovery from microarray data sets ^7^. The HotNet algorithm ^5, 6^ was implemented by porting python and MatLab code of HotNet _v1.0.0 (downloaded from http://compbio.cs.brown.edu/projects/hotnet/) to Java and R. For details about other algorithms and their implementations, please refer to our previous work ^3, 7^.

The majority of interactions in the Reactome FI network are extracted from reactions and complexes. In order to display semantic meanings (e.g. catalysis, activation and inhibition) of these interactions, we created a Reactome FI network specific visual style. This visual style is registered as a service using the OSGi API supported by Cytoscape 3, and applied to newly constructed FI sub-network automatically for network analysis.

### Pathway analysis features

Since version 4.0.0.beta, released in January 2014, ReactomeFIViz allows users to explore a list of high quality, human curated Reactome pathways, visualize Reactome pathways directly in Cytoscape, and perform pathway enrichment analysis on a list of genes based on a binomial test ^8^. In April 2014, we added a new experimental feature for performing integrated pathway analysis for multiple genomic data types by adapting a factor graph based approach called “PARADIGM” ^9^ into ReactomeFIViz.

**Figure 2.**
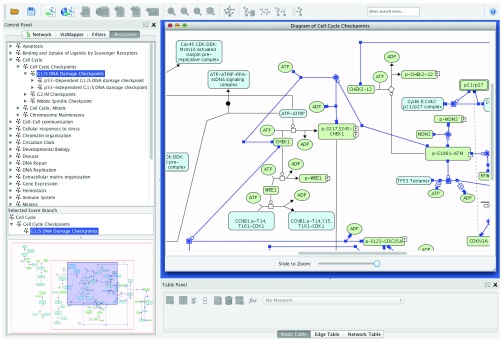
A Reactome pathway diagram displayed in a Reactome diagram view. The diagram view is wrapped in a JInternalFrame and hosted in the Cytoscape desktop.

The Reactome database contains several hundred manually laid-out pathway diagrams ^4^. Pathway diagrams in Reactome are drawn based on biochemical reactions. A reaction usually contains multiple inputs and outputs, in addition to catalysts, inhibitors and activators. The network model in Cytoscape is designed to support simple graphs containing edges between two nodes only. In order to display Reactome pathway diagrams, we adapted the pathway diagram view in the Reactome curator tool ^4^ into the Cytoscape environment, and wrapped it in a JInternalFrame so that a pathway view can be displayed along with a network view in the Cytoscape desktop ( Figure 2).

## Results

**Figure 3.**
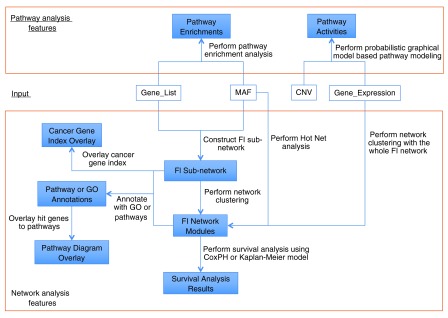
Major features implemented in ReactomeFIViz. Features implemented in ReactomeFIViz are roughly categorized into two types: pathway analysis features (top) and network analysis features (bottom). Input for these features can be a simple gene list contained in a text file, a mutation annotation file (MAF), a text file for gene expression data, or copy number variants (CNVs).

ReactomeFIViz provides a suite of features to assist users to perform pathway- and network-based data analyses ( Figure 3). Based on a list of genes loaded from a file, the user can construct a sub-network, perform network clustering to search for network modules related to patient clinical or other phenotypic information, annotate network modules, perform pathway enrichment analysis, and even model pathway activities based on probabilistic graphical models ^9^. By performing pathway- and network-based analyses using ReactomeFIViz, researchers will be able to uncover pathway and network patterns related to their studies and then link found patterns to clinical phenotypes ^3, 7^.

**Figure 4.**
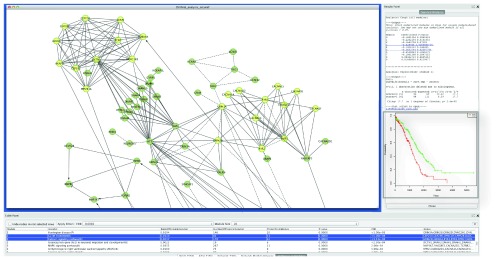
Module 3 generated from the TCGA ovarian cancer mutation data file is significantly related to patient overall survival. The central main panel shows the network view of module 3. The bottom table displays pathway annotations for genes in module 3 with two pathways, Calcium Signaling Pathway and G2/M Transition, highlighted. The right panel shows survival analysis results using both the Cox proportional hazards (CoxPH) model and the Kaplan-Meier model. The Kaplan-Meier plot was added to the figure later.

As an example, we present results generated from network module based analysis for the TCGA ovarian cancer mutation data ^10^ using ReactomeFIViz. The TCGA mutation data file and clinical information file were downloaded from the Broad Institute Firehose web site https://confluence.broadinstitute.org/display/GDAC, released in July 2012. The clinical information has been pre-processed.). For this data set, we chose the 2009 version of the FI network, and picked genes mutated in three or more samples to construct a FI sub-network. We performed a network clustering, followed by survival analysis for each network module by splitting samples into two groups: samples having genes mutated in the module (Group 1) and samples not having genes mutated in the module (Group 0). Our results indicate that group 1 samples ( Figure 4, green line in the Kaplan-Meier plot ^11^) have significantly longer overall survival times compared to group 0 samples ( Figure 4, red line in the Kaplan-Meier plot) (p-value = 3.4 × 10 ^-5 ^based on the CoxPH analysis ^12^) based on module 3. Pathway enrichment analysis results imply that module 3 is enriched with genes in calcium signaling pathway ( http://www.genome.jp/kegg/pathway/hsa/hsa04020.html) and mitotic G2/M transition ( http://www.reactome.org/cgi-bin/control_panel_st_id?ST_ID=REACT_2203.2). These results suggest that mutations impacting calcium signaling and the cell cycle may increase the survival of ovarian cancer patients. However, we may need more samples and independent data sets to validate our conclusion.

Using the same version of ReactomeFIViz but different versions of the FI network may yield different results because of updates of protein interactions in the FI network. We performed the same analysis with the latest version of the FI network (the 2013 version), and found that genes in module 3 from the 2009 version of the FI network have been split among several modules discovered using the newer version of the FI network. The module having the largest overlap with module 3 from the 2009 version of the FI network has the most significant p-value from the survival analysis (p-value = 1.1 × 10 ^-3^ from CoxPH), which implies that our method is fairly robust against updates of the FI network. For details, see the supplementary results.

## Discussion

Our Cytoscape app provides a suite of features for users to perform network- and pathway-based analysis for data generated from multiple experiments related to cancer and other complex diseases. Users can use our tool to search for disease-related network and pathway patterns. Our tool is built upon the Reactome database, arguably the most comprehensive human curated open source pathway database, and leverages the highly reliable functional interaction network extracted from human curated pathways. Many studies based on the FI network and this app have shown its many applications to cancer and other disease studies ^13– 16^.

For future development, we will focus on using probabilistic graphical models, such as factor graphs, for performing pathway modeling and linking results to patient clinical information in order to uncover cellular mechanisms related to cancer drug sensitivity, search for cancer biomarkers, and assist new drug development.

## Data availability

Data files used in the example: http://reactomews.oicr.on.ca:8080/caBigR3WebApp/TCGA_OV_Firehose_MAF_CLIN_2012.zip.

Use the detailed procedures described in our user guide to reproduce the results described in the example: http://wiki.reactome.org/index.php/Reactome_FI_Cytoscape_Plugin.

## Software availability

Homepage: http://wiki.reactome.org/index.php/Reactome_FI_Cytoscape_Plugin

Cytoscape app: http://apps.cytoscape.org/apps/reactomefiplugin

Latest source code: https://github.com/reactome-fi/CytoscapePlugIn

Source code as at the time of publication: https://github.com/F1000Research/CytoscapePlugIn

Archived source code as at the time of publication: http://www.dx.doi.org/10.5281/zenodo.10385 ^17^

License: the Creative Commons Attribution 3.0 Unported License ( http://www.reactome.org/?page_id=362).

## Figures and Tables

**Figure S1.**
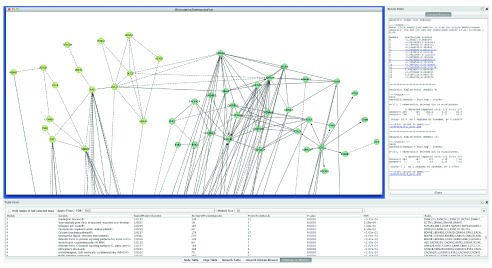
Modules 4 and 11 from the TCGA ovarian cancer mutation data file generated by using the 2013 version of the FI network. The central panel shows the network view of module 4 (right in lightseegreen) and module 11 (left in darkkhaki). The bottom table displays pathway annotations for module 4. The right panel shows survival analysis results using the CoxPH model for modules containing genes no less than 10 and the Kaplan-Meier model for modules 4 and 11.

**Figure S2.**
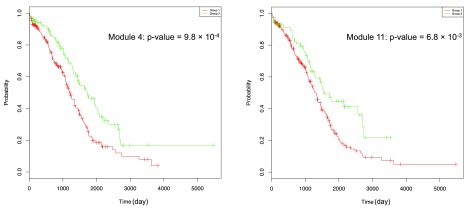
Kaplan-Meier survival plots for modules 4 and 11. P-values are calculated based on the Kaplan-Meier survival model.

**Table S1.** Distribution of module 3 genes from the 2009 version of the FI network into modules from the 2013 version of the FI network. Column Module is for module indices, Size for numbers of genes contained by modules from the 2013 version of the FI network, Shared for numbers of genes shared between 2009 module 3 and 2013 module, and P-value for significance of sharing based on the hypergeometric test.

Module	Size	Shared	P-value
4	30	19	5.5E-13
3	60	21	5.8E-08
26	2	2	1.2E-02
11	16	5	2.2E-02
1	97	4	1.0E+00
0	110	1	1.0E+00

## Supplementary results

In this supplementary document, we describe analysis results for the example data set using the 2013 version of the FI network. Figure S1 shows two modules, module 4 and module 11, having smallest p-values from survival analyses based on the CoxPH model. Figure S2 shows the Kaplan-Meier plots for Modules 4 and 11. Overlapping analysis ( Table S1) indicated that 58 genes in the module 3 from the 2009 version of the FI network have been spread into module 4 (18 genes, p-value = 5.5 × 10 ^-13^ based on hypergeometric test), module 3 (21 genes, p-value = 5.8 × 10 ^-8^), module 26 (2 genes, p-value = 0.01), module 11 (5 genes, p-value = 0.02), module 1 (4 genes, p-value = 1.0), and module 0 (1 gene, p-value = 1.0). It is interesting to see that module 4 has the most significant overlap with module 3 from the 2009 version of the FI network, and also has the most significant p-value from the survival analyses (p-value = 1.1 × 10 ^-3^ from CoxPH), which implies that our method is fairly robust against updates of the FI network.
