# Supplementary material for: ReactomeFIViz: a Cytoscape app for pathway and network-based data analysis
Source: F1000Res. 2014 Sep 12;3:146. Originally published 2014 Jul 1. [Version 2] doi: 10.12688/f1000research.4431.2 (PMC4184317; doi:10.12688/f1000research.4431.2)
Supplement: Peer review history

# Peer review: Referee response for version 2

**DOI:** 10.5256/f1000research.5631.r6104

**Published:** 2014-09-22

**Authors:** Nikolaus Schultz, B. Arman Aksoy

**Affiliations:** 1 Computational Biology Center, Memorial Sloan-Kettering Cancer Center, New York, USA; 2 Memorial Sloan Kettering Cancer Center, New York, USA

The authors have addressed all of our comments and concerns.

We have read this submission. We believe that we have an appropriate level of expertise to confirm that it is of an acceptable scientific standard.

# Peer review: Referee response for version 1

**DOI:** 10.5256/f1000research.4742.r5319

**Published:** 2014-08-11

**Authors:** Nikolaus Schultz, B. Arman Aksoy

**Affiliations:** 1 Computational Biology Center, Memorial Sloan-Kettering Cancer Center, New York, USA; 2 Memorial Sloan Kettering Cancer Center, New York, USA

In this manuscript, the authors describe details of a Cytoscape 3.0 App: ReactomeFIViz. This app is intended to help users easily investigate genomic alteration data in the context of biological networks. The app allows users to obtain a network from the curated Reactome Functional Interaction database; map mutation, copy-number alteration or gene expression data onto the network; conduct a gene set enrichment analysis or module discovery on the simplified Reactome network; and finally, see the detailed pathway view provided by the Reactome Pathway Browser.

The previous version of the App, which was compatible with Cytoscape 2, was introduced as a supplemental software tool in an earlier study (Wu, Feng and Stein, 2010). Since then, both Cytoscape extensions, the plug-in and the app, have actively been used by users. This has also been evident by the positive reviews the App has received on the Cytoscape App Store. Considering the rich functionality and the ease of cancer genomics analysis that the App provides to the users, we believe this app is of interest to many researchers working in the fields of Computational Biology, Cancer Genomics and Systems Biology.

There is already extensive documentation about the App on the Reactome web site, however the manuscript fails to provide a general overview for non-experienced users. We have the following suggestions for the authors and if addressed, we believe, these will considerably improve the manuscript:

- In the abstract, the authors say “ *... pathways from Reactome and other pathway databases...*“. The paper, however, creates the impression that the App is highly dependent on the Reactome infrastructure and does not allow communication with other databases. We suggest that the authors remove *“and other pathway databases*” from the abstract or better clarify this point in the abstract.
- The details about the type of analyses and the statistical tests the App enables are provided in an earlier paper by the same group (Wu, Feng and Stein, 2010), but not in this paper. For readers that are interested in learning these details, we suggest the authors to add a sentence to the manuscript and refer to their earlier work for details. If the functionality and the implementation of these tests have changed since then, we suggest that the authors clearly list these new improved items in the paper for more clarification.
- As an example use case, the authors provide the details of a re-analyzed data set and mention that the results of these two analyses differ due to changes in Reactome FI networks. Curated databases are, of course, subject to changes over time; but it is not clear from the text whether it was the changed network that was causing the problem or the new version of the extension. We suggest that the authors provide the results of these two runs as a supplement to their paper for users to compare and contrast.
- The last sentence of the *Implementation *section says that the analyses were conducted in R, but can the authors clarify the requirements for this App? Do users, for example, need to install R to use this App? Related to this, the authors do not talk about the Cytoscape version that the App targets. Is the Cytoscape 2 PlugIn deprecated? Do authors suggest that users install the newer version in Cytoscape 3 as an App?
- Figure 1 provides details about the implementation and the architecture of the ReactomeFIViz App, however we think the manuscript needs a simple user flow diagram that shows where different types of data are obtained, the functionalities of the App and the output the users get. The mutation-based module discovery and differential survival analysis examples mentioned in the paper are good use cases, however it is not clear from the text what the App supports other than these examples.
- Finally, we suggest the authors to provide a supplemental step-by-step guide to replicate the results that they describe in the paper. For a new user, this may provide a good base to start using the software and for many researchers it might be more convenient to have such an article provided as a supplementary file to this paper.

We have read this submission. We believe that we have an appropriate level of expertise to confirm that it is of an acceptable scientific standard, however we have significant reservations, as outlined above.

# Response

**Published:** 2014-09-06

**Authors:** Guanming Wu

Thanks a lot for your review of our article. We have made changes according to your thoughtful suggestions. Please see details below:

 

- *In the abstract, the authors say “... pathways from Reactome and other pathway databases...“. The paper, however, creates the impression that the App is highly dependent on the Reactome infrastructure and does not allow communication with other databases. We suggest that the authors remove “and other pathway databases” from the abstract or better clarify this point in the abstract.*
  Although the app uses a software infrastructure running on Reactome, the curated pathways it uses for the network and pathway enrichment analysis come in equal parts from Reactome and other pathway databases. We have clarified this in the abstract.

- *The details about the type of analyses and the statistical tests the App enables are provided in an earlier paper by the same group (Wu, Feng and Stein, 2010), but not in this paper. For readers that are interested in learning these details, we suggest the authors to add a sentence to the manuscript and refer to their earlier work for details. If the functionality and the implementation of these tests have changed since then, we suggest that the authors clearly list these new improved items in the paper for more clarification.*
  We have added references to our original FI network paper in the paragraph introducing “Network analysis features”. Also, we added the date that version 4.0.0.beta was released in order to indicate that pathway analysis features are new features that have not been covered by our previous work.
- *As an example use case, the authors provide the details of a re-analyzed data set and mention that the results of these two analyses differ due to changes in Reactome FI networks. Curated databases are, of course, subject to changes over time; but it is not clear from the text whether it was the changed network that was causing the problem or the new version of the extension. We suggest that the authors provide the results of these two runs as a supplement to their paper for users to compare and contrast.*
  We modified the sentence in the last paragraph describing different results using different versions of the FI network to make it clear that the same software, but different versions of the FI network, were used. We also deleted this sentence in the implementation section, “Each version is handled by its own web application on the server-side for easy software maintenance.”, to avoid confusing the readers with technical details.
  As suggested, added a supplementary document to describe results from the 2013 version of the FI network. 
- *The last sentence of the Implementation section says that the analyses were conducted in R, but can the authors clarify the requirements for this App? Do users, for example, need to install R to use this App? Related to this, the authors do not talk about the Cytoscape version that the App targets. Is the Cytoscape 2 PlugIn deprecated? Do authors suggest that users install the newer version in Cytoscape 3 as an App?*
  We have clarified the section to make the requirements clearer, and added a new paragraph to address the relationship of the application to Cytoscape 2 and 3.
- *Figure 1 provides details about the implementation and the architecture of the ReactomeFIViz App, however we think the manuscript needs a simple user flow diagram that shows where different types of data are obtained, the functionalities of the App and the output the users get. The mutation-based module discovery and differential survival analysis examples mentioned in the paper are good use cases, however it is not clear from the text what the App supports other than these examples.*
  We have added a new paragraph at the top of the Results section to highlight some major features in the App, and created a new diagram to show these major features as suggested.
- *Finally, we suggest the authors to provide a supplemental step-by-step guide to replicate the results that they describe in the paper. For a new user, this may provide a good base to start using the software and for many researchers it might be more convenient to have such an article provided as a supplementary file to this paper.*
  The application’s web-based tutorial already has provided very detailed, step-by-step instructions that replicate the results. We have added a new sentence in the Data availability section to point this out, and the README file contained in the zip file for downloading now points readers interested in replicating the analysis to the online tutorial.

# Peer review: Referee response for version 1

**DOI:** 10.5256/f1000research.4742.r5320

**Published:** 2014-07-23

**Authors:** Hamid Bolouri

**Affiliations:** 1 Division of Human Biology, Fred Hutchinson Cancer Research Center, Seattle, WA, USA

This article describes version 4.1.0 of the Reactome Functional Interaction "plugin"/"app" for the Cytoscape network exploration/analysis Java platform. 

The Reactome Functional Interaction network is an established, well-received and widely used resource. The software described here provides useful resources both for exploring Reactome networks and for using them in network enrichment analyses.

My only reservation about this article is that its brevity makes it sketchy. For example, how are pathway enrichment statistics calculated? Does the implementation of HotNet offered here differ in any way from the original?

I am especially intrigued that the example use-case given is reported to work well with the 2009 version of the ReactomeFI network, but apparently the discovered modules cannot be found with the newer versions of the FI because the underlying interactions have been "s *pread into several modules in the newer version of the FI network*". Why does this happen and how can users guard against it?

I was also not able to explore/reproduce the example presented in the paper, because (contrary to its ".txt"extension) the MAF file provided did not appear to be plain text and the needed survival data file was not included in the zip file provided. A 'README' file with instructions may be useful here.

P.S. The multiple names given to this resource - "ReactomeFIViz (also called the Reactome FI Cytoscape app or ReactomeFIPlugIn)" - are a little confusing and seem unnecessary.

I have read this submission. I believe that I have an appropriate level of expertise to confirm that it is of an acceptable scientific standard.

# Response

**Published:** 2014-09-06

**Authors:** Guanming Wu

Thanks a lot for your comments. We have made changes according to your comments. Please see details below:

- *My only reservation about this article is that its brevity makes it sketchy. For example, how are pathway enrichment statistics calculated? Does the implementation of HotNet offered here differ in any way from the original?*
  We have now stated that a binomial test is used for pathway enrichment analysis in the “Pathway analysis features” section and cited a reference (reference 8) for it. We also added a sentence to point out that our implementation of HotNet was done by porting the original Python and MatLab code to Java and R, and so the original algorithm is unchanged. 
- *I am especially intrigued that the example use-case given is reported to work well with the 2009 version of the ReactomeFI network, but apparently the discovered modules cannot be found with the newer versions of the FI because the underlying interactions have been "spread into several modules in the newer version of the FI network". Why does this happen and how can users guard against it?*
  Because new interactions have been added into the latest version of the FI network, these new interactions may change the network clustering results. However, we still find some network modules, which overlap significantly with the original module and have significant p-values, though higher than the original one, from survival analysis. As suggested by another reviewer, we have added a supplementary document to describe results from the 2013 version of the FI network. As stated in the manuscript, we recommend that the user should use the latest version of the FI network, and check with previous versions of the FI network to investigate the stability of found network modules.
- *I was also not able to explore/reproduce the example presented in the paper, because (contrary to its ".txt"extension) the MAF file provided did not appear to be plain text and the needed survival data file was not included in the zip file provided. A 'README' file with instructions may be useful here.*
  We apologize for the problem. It turned out that the zip file was collapsed somehow. We have fixed the problem, and added a simple README file as suggested.
- *The multiple names given to this resource - "ReactomeFIViz (also called the Reactome FI Cytoscape app or ReactomeFIPlugIn)" - are a little confusing and seem unnecessary.*
  Because of the development history of this software, the same app has been called different names. For example, prior to Cytoscape 3, all Cytoscape extensions were called “plug-ins”, but the nomenclature has now been changed to “app”. We have tried to minimize the confusion by using ReactomeFIViz consistently and referring to the names of earlier versions of the software just once. 
